# Fluid-Structure Interaction Based Algorithms for IOP and Corneal Material Behavior

**DOI:** 10.3389/fbioe.2020.00970

**Published:** 2020-08-28

**Authors:** Osama Maklad, Ashkan Eliasy, Kai-Jung Chen, JunJie Wang, Ahmed Abass, Bernardo Teixeira Lopes, Vassilis Theofilis, Ahmed Elsheikh

**Affiliations:** ^1^School of Engineering, University of Liverpool, Liverpool, United Kingdom; ^2^Eye Hospital and The Institution of Ocular Biomechanics, Wenzhou Medical University, Wenzhou, China; ^3^Beijing Advanced Innovation Center for Biomedical Engineering, Beihang University, Beijing, China; ^4^NIHR Biomedical Research Centre for Ophthalmology, Moorfields Eye Hospital NHS Foundation Trust and UCL Institute of Ophthalmology, London, United Kingdom

**Keywords:** ocular biomechanics, intraocular pressure, fluid-structure interaction (FSI), corneal material behavior, cornea

## Abstract

**Purpose:** This paper presents and clinically validates two algorithms for estimating intraocular pressure (IOP) and corneal material behavior using numerical models that consider the fluid-structure interaction between the cornea and the air-puff used in non-contact tonometry.

**Methods:** A novel multi-physics fluid-structure interaction model of the air-puff test was employed in a parametric numerical study simulating human eyes under air-puff pressure with a wide range of central corneal thickness (CCT = 445–645 μm), curvature (*R* = 7.4–8.4 mm), material stiffness and IOP (10–25 mmHg). Models were internally loaded with IOP using a fluid cavity, then externally with air-puff loading simulated using a turbulent computational fluid dynamics model. Corneal dynamic response parameters were extracted and used in development of two algorithms for IOP and corneal material behavior; fIOP and fSSI, respectively. The two algorithms were validated against clinical corneal dynamic response parameters for 476 healthy participants. The predictions of IOP and corneal material behavior were tested on how they varied with CCT, R, and age.

**Results:** The present study produced a biomechanically corrected estimation of intraocular pressure (fIOP) and a corneal material stiffness parameter or Stress-Strain Index (fSSI), both of which showed no significant correlation with R (*p* > 0.05) and CCT (*p* > 0.05). Further, fIOP had no significant correlation with age (*p* > 0.05), while fSSI was significantly correlated with age (*p* = 0.001), which was found earlier to be strongly correlated with material stiffness.

**Conclusion:** The present study introduced two novel algorithms for estimating IOP and biomechanical material behavior of healthy corneas *in-vivo*. Consideration of the fluid structure interaction between the cornea and the air puff of non-contact tonometry in developing these algorithms led to improvements in performance compared with bIOP and SSI.

## Introduction

It is of increasing clinical importance to quantify the biomechanical properties of the cornea *in vivo*. It would allow better evaluation of corneal ectatic diseases such as keratoconus (KC) (Ye et al., 2015; Bao et al., 2016; Chang et al., 2018) and enable customization of procedures that interact or interfere mechanically with the cornea including refractive surgeries (Sorsby, 1953; Dhaliwal, 1985), collagen cross-linking treatment (Spoerl et al., 2011; Chang et al., 2018), and intrastromal corneal ring segment (ICRS) implantation (Roberts and Dupps, 2014).

The estimation of IOP is an essential measurement in eye examination and crucial in monitoring and treatment of ocular pathologies including glaucoma and ocular hypertension (Stamper, 2011). Therefore, accurate estimation of IOP is highly desirable as the risk of glaucoma progression rises by 11% for every 1 mmHg increase in IOP (Bengtsson et al., 2007). The gold standard of IOP measurements is the Goldmann Applanation Tonometer (GAT), which apply a contact force to a central area of the cornea and when this area flattens, it assumes that the external applied pressure equals the internal IOP (Goldmann and Schmidt, 1957). This measurement technique makes IOP values sensitive to the natural variations in the central corneal thickness (CCT) and stiffness of the corneal tissue and introduces unacceptable inaccuracies (Hemdon et al., 1997; Liu and Roberts, 2005; Eliasy et al., 2018). This was the main motivation for several attempts to provide IOP estimates that are corrected for corneal biomechanics, such as the Ocular Response Analyzer (ORA Reichert Ophthalmic Instruments, Depew, NY) (Luce, 2005), (Montard et al., 2007), and the CorVis ST (OCULUS Optikgeräte GmbH; Wetzlar, Germany) (Joda et al., 2015; Vinciguerra et al., 2016a). These two devices use a puff of air to applanate the central part of the cornea, where ORA used the cornea's two applanation pressure to reduce association of IOP with CCT and developed the cornea-corrected IOP (IOPcc) estimate, while CorVis-ST uses a high speed Scheimpflug imaging to trace deformation of both the cornea's anterior and posterior profiles under effect of the external air pressure. This high speed imaging technique enabled accurate measurement of corneal thickness, curvature, and corneal deformation patient-specific parameters, which allowed reliable representation of corneal behavior in numerical modeling to produce the bIOP estimation algorithm.

Here comes the benefit of the *in vivo* corneal biomechanical characterization in obtaining more accurate estimates of intraocular pressure (IOP) (Elsheikh et al., 2015). The non-linearity of corneal tissue behavior makes the determination of the behavior *in vivo* quite challenging as the gradient of the stress-strain curve (known as the tangent modulus, E_t_) is not constant but increases gradually with applied stress or pressure (Kotecha et al., 2006; Elsheikh et al., 2007; Elsheikh, 2010). This characteristic creates a difficult challenge with E_t_ (the measure of stiffness) being dependent on IOP, while the measurement of IOP using tonometry is affected by corneal stiffness. The challenge is to overcome this apparent inter-dependence and produce reliable estimates of both corneal stiffness and IOP.

Progress has been made recently in producing a biomechanically-corrected IOP (bIOP) estimate that is intended to be independent of corneal stiffness (Eliasy et al., 2018). A Stress-Strain Index (SSI) was also developed to estimate the cornea's stress-strain behavior, and hence E_t_ at any stress or IOP level (Eliasy et al., 2019). Both bIOP and SSI relied on the dynamic deformation parameters obtained in response to the rapid air-puff of the Corvis ST (OCULUS Optikgeräte GmbH; Wetzlar, Germany) (Ambrósio et al., 2017). Earlier studies have shown that bIOP was less influenced by corneal stiffness than both the Goldmann Applanation Tonometer (GAT) and the uncorrected Corvis readings (CVS-IOP) (Eliasy et al., 2018). The studies also found SSI to be almost independent of both central corneal thickness (CCT) and bIOP, while strongly correlated with age.

The present study intended to eliminate an important simplification made in the numerical analyses that led to the development of both bIOP and SSI, namely the assumption that the pressure caused by the air-puff maintained a constant distribution throughout all deformation stages. This assumption is eliminated in the numerical analyses conducted in this study through modeling a 3D air-puff impinging on the cornea using a turbulent computational fluid dynamics (CFD) and Arbitrary Lagrangian-Eulerian (ALE) deforming mesh to couple with the finite element model of the eye (Maklad, 2019; Maklad et al., 2020). This method allowed fluid-structure interaction between the air-puff and the eye and enabled the air pressure distribution on the eye to vary in response to corneal deformation in a stepped approach. The corneal deformation predictions obtained with the coupled models were then analyzed to develop new algorithms for bIOP and SSI that consider fluid-structure interaction, hence named fIOP and fSSI.

## Methods

### Numerical Models

**Figure 1:**
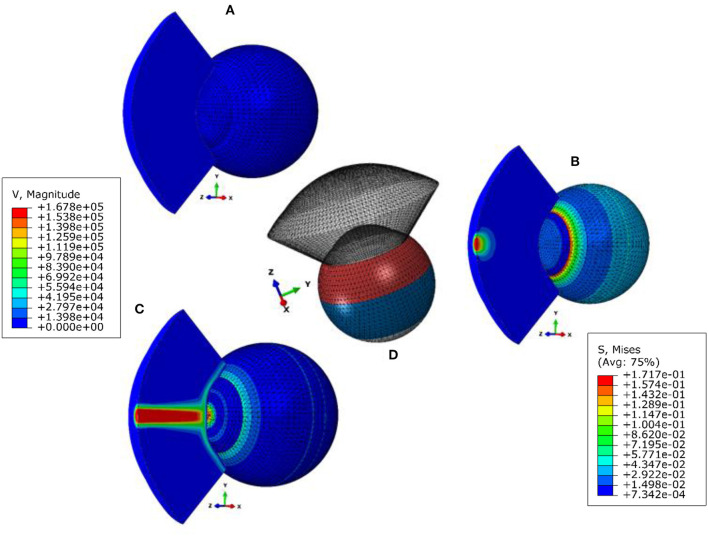
Fluid structure interaction (FSI) model of the air puff test **(A)** stress-free configuration, **(B)** model after applying internal IOP, **(C)** model at highest concavity, and **(D)** is showing the different material sections in the eye model. The legend on the left is the magnitude of the air velocity in mm/s from the CFD model and the legend on the right is the magnitude of Von-Mises stress in MPa from the finite element model of the eye.

This study was based on a novel multi-physics, fluid-structure interaction (FSI) model of the air-puff test of the Corvis ST on full eye globes subjected to the internal load of IOP. Details of the numerical model, including, its validation and the used FSI two-way coupling approach with all the co-simulation control parameters and equations were published in our earlier study Maklad et al. (2020). Here, we are giving the most important information, the air-puff was simulated using the turbulent Abaqus/CFD solver (version 6.14-2, Dassault Systèms Simulia Inc., USA) coupled with the finite element model of the eye using an arbitrary Lagrangian-Eulerian (ALE) deforming mesh, Figure 1. A mesh dependence study was performed and Figure S1 shows the apical deformation against number of eye model number of nodes and the pressure on Apex against the air model number of nodes along with the simulation running time, and based on this study the suitable number of elements were selected for every model.

The eye model consisted of 10,000 fifteen-nodded continuum elements (C3D15H) arranged in two layers to keep a consistent aspect ratio of the elements' dimensions, see Figure S2 in the Supplementary Material. Models of the air domain consisted of 103,680 six-nodded 3D fluid continuum elements (FC3D6) and used Spalart–Allmaras turbulent eddy viscosity model (Versteeg and Malalasekera, 1995; NASA, 2011) to simulate the turbulence in the air jet. To avoid excessive distortion of the air domain mesh during the coupling process with the eye model, an adaptive Arbitrary Lagrangian–Eulerian (ALE) deforming mesh was used to improve the stability of the simulation analysis (Hron and Turek, 2006; Kcharik et al., 2006; Donea et al., 2017). The finite element model of the eye was prevented from rigid body motion in the Z-direction (anterior-posterior) at the equatorial nodes. Also, the posterior pole node was restricted in both X and Y directions but were free to move in the Z-direction (anterior-posterior), see Figure S2 in the Supplementary Material. While the air model domain and its mesh were created over the cornea and a 4 mm ring of the sclera by projecting coordinates of the anterior surface nodes for a distance of 11 mm as this was the distance from the air puff nozzle to the corneal apex. The air jet inlet diameter was set to 2.4 mm, as given by the manufacturers for the nozzle of CorVis-ST, and the maximum air velocity at the inlet was set to 167.8 m/s, which corresponds to a maximum Reynolds number of 2.3 × 10^4^ (Maklad et al., 2020).

Models were generated with an anterior shape factor of 0.82, a limbal radius of 5.85 mm, a sclera external radius of 11.5 mm and the thickness regional variation reported in earlier studies (Kotecha et al., 2006; Elsheikh et al., 2007, 2010a; Eliasy et al., 2019). The eye models adopted the material stress-strain behavior patterns that were found in earlier experimental studies to correlate with age within the range 30–100 years (Elsheikh et al., 2010a,b; Eliasy et al., 2019). They were as presented in Equation 1 for corneal material, and Equation 2 for anterior, equatorial and posterior sclera.

(1) $$\begin{array}{l} \sigma =\left(35\times 10^{-9}age^{2}+1.4\times 10^{-6}age+1.03\times 10^{-3}\right)\\ \times \left[e^{\left(0.0013age^{2}\;+\;0.013age\;+\;99\right)\varepsilon }-1\right] \end{array}$$

(2) $$\begin{array}{r} \sigma =\frac{2\mu }{\alpha }.\left({\left(\varepsilon +1\right)}^{\alpha -1}-{\left(\varepsilon +1\right)}^{-\left(1+\frac{\alpha }{2}\right)}\right), \end{array}$$

$$\begin{array}{l} where\;\{\begin{array}{c} \mu =1.26\;age+0.94,\;\alpha =20.1\;age+19.8,\;for\;anterior\;sclera\\ \mu =0.85\;age+0.42,\;\alpha =12.6\;age+34.16,\;for\;equatorial\;sclera\\ \mu =0.22\;e^{1.19\;age}\;,\;\alpha =53.02,\;for\;posterior\;sclera \end{array} \end{array}$$

In Equations 1 and 2, σ is the stress in MPa and age is in years. Similar to the SSI (Eliasy et al., 2019), fSSI was set to 1.0 for a stress-strain relationship that corresponded to the mean behavior found experimentally for corneas aged 50 years (Elsheikh et al., 2010b). The patient's age is a direct parameter in changing corneal material stiffness which is a crucial input parameter in the material definition for the finite element model of the eye according to the experimental study conducted by Elsheikh et al. (2010b). Increases and decreases in fSSI relative to 1.0 corresponded to stress-strain relationships for which E_t_ at any stress level grew or reduced by the same change in fSSI. With this principle in mind, the stress-strain behavior in the cornea that corresponded to any age could be converted into an fSSI value.

**Figure 2:**
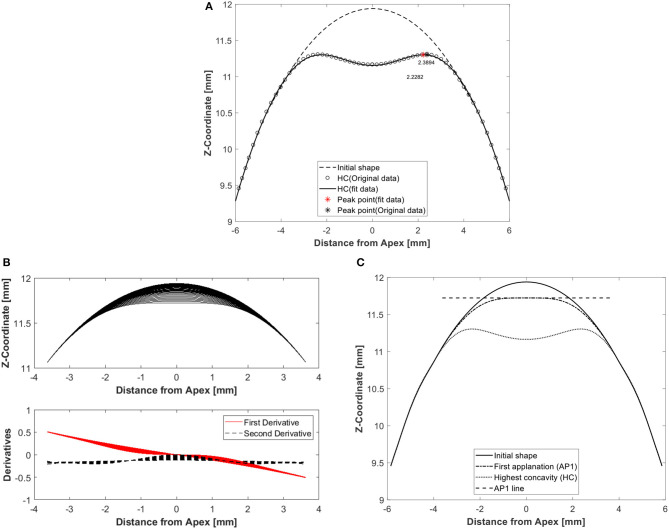
Example results of a typical analysis showing corneal deformation parameters from the numerical model including **(A)** peak point location at highest corneal concavity to calculate peak distance, which is the distance between the two corneal peaks, **(B)** first applanation moment determined by using first and second derivatives of corneal profiles over the 7 mm diameter central zone, and **(C)** applanation length by calculating the difference between Apex Z-coordinate and its neigbouring points until a tolerance of 0.01 is broken.

The eye models were built using a bespoke software package generated in MATLAB® (Natick, Massachusetts, USA). The analysis started by finding the stress-free geometry (under zero IOP) for each eye model using an iterative approach reported earlier (Elsheikh et al., 2013). The coupled models were then subjected to IOP followed by Corvis air pressure, and the resulting deformation across the eye globe was stored for later analysis. Another bespoke MATLAB code was used to extract and record the corneal response parameters, example is shown in Figure 2, along with the models' input parameters (Roberts, 2017; Jedzierowska and Koprowski, 2019; Maklad, 2019). Figure 2A shows the peak point location at highest corneal concavity, at which stage the peak distance was calculated as the distance between the two corneal peaks. This calculation started with fitting the corneal curve to a polynomial, identifying the points with maximum Z-coordinate and finding the distance from corneal center (X-coordinate). Figure 2B shows the method used to determine the time to first applanation, and the corresponding air pressure and apical deformation. This was done by calculating the first and second derivatives of the corneal profile at apex for every time step. When the derivatives reach a value of zero, indicating a flattened corneal surface, this behavior stage was considered the point of first applanation. On the other hand, Figure 2C illustrates how the applanation length is estimated by calculating the difference between the Z-coordinates at apex and neighboring points. Where the difference in Z-coordinate exceeded 0.01 mm was considered the end of the peak length. A parametric study was carried out to gauge the influence of model input parameters on corneal biomechanical parameters as a response to the air-puff. This was performed with wide ranges of central corneal thickness, CCT, between 445 and 645 μm, IOP between 10 and 25 mmHg, central corneal curvature, R, between 7.4 and 8.4 mm, and corneal material stiffness coefficient, μ (stiffness parameter), between 0.0422 and 0.1082. The influence matrix of each parameter on corneal response parameters is shown in Figure S3 in Supplementary Material and all Pearson's correlation values are shown in Table S1, which was published in our earlier study, Maklad et al. (2020).

### fIOP and fSSI Algorithms

The numerical models had IOP, CCT, R, and age as input parameters, and the output was corneal deformation profiles that resulted in response parameters including, most notably, peak distance, first applanation deformation amplitude, first applanation length, highest concavity deformation amplitude and highest concavity radius, Figure 2. Analysis of the input and output parameters allowed the development of two relationships for fIOP as a function of CCT, R, age, and corneal deformation parameter with strong correlation with true IOP and fSSI as a function of CCT, fIOP, and corneal deformation parameter with strong correlation with material stiffness.

The significance of the correlations of corneal deformation parameters with IOP and age was assessed in SPSS Version 24 (IBM Corp., USA) and confirmed with a probability value, *p* < 0.05 or a high Pearson's correlation coefficient (*r*). For each of the parameters for which significant correlation was confirmed, an exercise was conducted to determine the lowest possible polynomial order that should be adopted in the fIOP equation based on the lowest route mean square error (RMSE). The objective function adopted took the form:

(3) $$\begin{array}{r} Objective\;function=\min RMS=\min\sqrt{\frac{1}{N}\;\overset{N}{\underset{i\;=1}{\sum }}{\left(fIOP_{i}-true\text{\_}{IOP}_{i}\right)}^{2}}, \end{array}$$

Where *RMS* is the root mean square of the error, *N* is the number of eye models, and *true_IOP* is the value set in the numerical models.

The development of the fSSI followed a similar route and the objective function used took the form:

(4) $$\begin{array}{r} Objective\;function=\min RMS=\min\sqrt{\frac{1}{N}\;\overset{N}{\underset{i=1}{\sum }}{\left(fSSI-SSI\right)}^{2}}, \end{array}$$

Where *RMS* is the root mean square of the error, *N* is the number of eye models, and *SSI* is the value set in the numerical models.

### Clinical Validation

In this retrospective study, we reviewed the Corvis data of right and left eyes of 476 healthy participants from the Vincieye Clinic in Milan, Italy and Rio de Janeiro Corneal Tomography and Biomechanics Study Group, Brazil. The participants had an age range between 10 and 87 years, CCT between 455 and 630 μm and IOP between 9 and 25 mmHg, Table 1. The data included the maximum deformation, first applanation pressure, first applanation time, highest concavity radius, spatial and temporal corneal deformation. The Institutional review board of the University of Liverpool ruled that approval was not obligatory for this record review study. However, the ethical standards set out in the 1964 Declaration of Helsinki and their revision in 2013 were observed and all patients provided informed written consent before using their de-identified data in research.

**Table 1:** Clinical dataset used in the validation of fIOP and cornea material characterization algorithms.

**Datasets**	**Participants**	**Age (years)**	**CCT (μm)**	**CVS-IOP (mmHg)**
Dataset 1 (Milan)	225	38 ± 17.2 (7–91)	543 ± 31.5 (458–635)	15.7 ± 2.35 (11–25)
Dataset 2 (Rio)	251	43 ± 16.5 (8–87)	539 ± 33.2 (454–629)	14.8 ± 3.06 (6–34)

The data was used to assess whether, as expected, fIOP was independent of CCT, age, and R. Similarly, fSSI's independence of CCT and IOP, and correlation with age were assessed using the same dataset. This exercise also enabled comparing fIOP against bIOP, and fSSI against SSI, in order to check whether the improved modeling adopted in this study, through consideration of the fluid-structure interaction, had led to improvements in IOP and material stiffness estimates.

## Results

### Air Pressure Distribution

**Figure 3:**
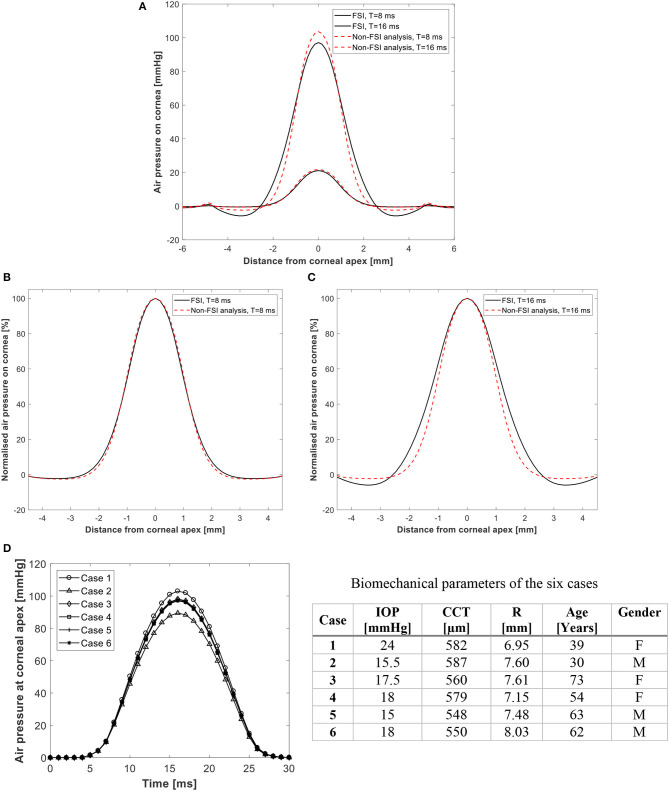
Predicted pressure distribution on the cornea with and without FSI analysis in **(A)** actual values, and **(B,C)** normalized values at 8 and 16 ms after start of pressure application. Temporal pressure profiles for 6 different models are shown in **(D)**. Details of the 6 cases are given in table.

To demonstrate the effect of fluid-structure interaction on the value and distribution of air pressure acting on the cornea, the results of two typical simulations are first compared; one assuming a rigid cornea that does not change shape under air pressure and another with FSI coupling between the air domain and the finite element model of the eye. Figures 3A–C shows the two pressure distributions as actual and normalized values at times *T* = 8 and 16 ms and demonstrate a small reduction in apical pressure of around 6.3% at 16 ms when FSI was considered. Additionally, Figure 3D shows how the temporal pressure profile changes from one model to another due to changes in the corneal biomechanical parameters. The means of these differences were small 3.4% at *T* = 8 ms and increased to 8.4% at 16 ms. Figure S4 in Supplementary Material shows the air velocity and pressure coefficient distribution on the cornea explaining how the dynamic pressure converts into static pressure on the cornea and why there is a negative pressure region at 2 mm from cornea apex.

### Correlation Analysis

A bivariate correlation analysis was carried out between each of the model's output corneal response parameters and the four main input parameters (IOP, CCT, R and age – representing corneal stiffness) and the influence matrix of each parameter on corneal response parameters is shown in Figure S2 in the Supplementary Material. The full correlation analysis and comparison with the clinical corneal response parameters are available in our earlier study, Maklad (2019), Maklad et al. (2020), which revealed that the first applanation pressure (AP1), and the highest concavity radius (R_HC_) were the highest correlated parameters to IOP (*r* = 0.736 and 0.624, respectively, and *p* < 0.001). For this reason, AP1 and R_HC_, along with CCT, R, and age were included in the fIOP equation. On the other hand, the stiffness parameter at highest concavity (SP- HC) was the most associated response parameters to corneal material change (*r* = 0.442, *p* < 0.01), and was therefore included with CCT and fIOP in the corneal material estimation algorithm.

### fIOP Equation

Using the least-squares method, the fIOP equation took the form:

(5) $$\begin{array}{r} fIOP\;=\;C_{AP1}\;\times C_{CCT-age}\times C_{R}\times C_{R_{HC}}+\;C \end{array}$$

where

*C*_*AP*1_ = (−0.005 × *AP*1+0.19)

*C_CCT-age_* = (0.011 × *CCT*^3^ μ^3^ − 0.002 × *CCT*^3^ μ^2^ + 9.17 × *CCT*^3^ μ + 8.34 × *CCT*^3^ − 6.3 × *CCT*^2^ μ^3^ + 1.16 × *CCT*^2^ μ^2^ − 0.05 × *CCT*^2^ μ − 0.003 × *CCT*^2^ + 0.76 × *CCT* μ^3^ + 5.67 × *CCT* μ^2^ − 4.87 × *CCT* μ + 1.73 × *CCT* − 0.55 × μ^3^ + 0.76 × μ^2^ + 1.82 × μ + 4.09)

μ = (0.076 *e*^0.536 *age*^)

*C_R_* = (0.045 × *R* − 0.213 × 10^−3^)

*C*_*R*_*HC*__ = (−0.0008 × *R*_*HC*_ − 0.68)

*C* = 9.36

In this equation, fIOP and AP1 were in mmHg, CCT in microns, R and R_HC_ in mm and age in years. With this equation form, the RMS error was 4.5%.

### Validation of fIOP Using Clinical Data

**Figure 4:**
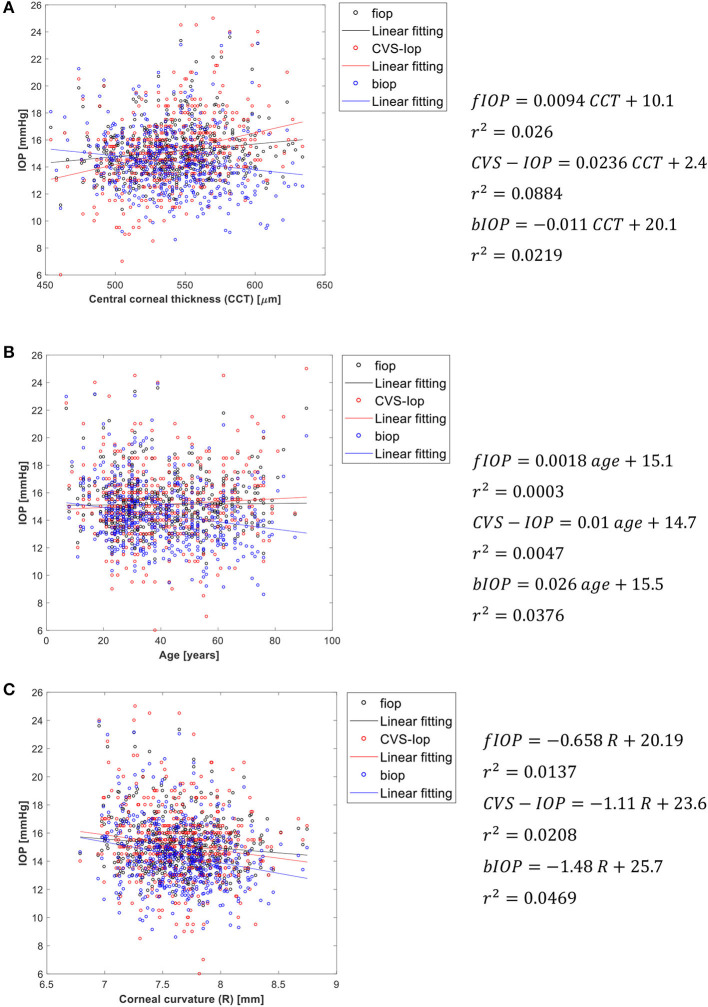
Association of fIOP, CorVis, and bIOP values with **(A)** central corneal thickness, **(B)** age, and **(C)** corneal apical radius.

Figure 4 presents an analysis of the association of fIOP, the previously developed bIOP, and the uncorrected Corvis IOP readings (CVS-IOP) with CCT, age and corneal curvature. The results show similar performance of fIOP with that of bIOP in reducing the association CVS-IOP with CCT. The figure also demonstrates better performance with fIOP than with bIOP in reducing the association of CVS-IOP with both age and R.

### Material Stiffness (fSSI) Algorithm

Similar to the fIOP equation, the fSSI algorithm was developed using the least-squares method, leading to the following form:

(6) $$\begin{array}{l} fSSI=\{\begin{array}{c} 0.5,\;for\;Ln\left(SP-HC\right)=0.026\;+\;1.83\;\times \left(fIOP/20\right)\;+\;2.26\;\times \;\left(CCT/545\right)\\ 1.0,\;for\;Ln\left(SP-HC\right)=0.68\;+\;1.44\;\times \left(fIOP/20\right)\;+\;2.36\;\times \;\left(CCT/545\right)\\ 1.5,\;for\;Ln\left(SP-HC\right)=0.85\;+\;1.49\;\times \left(fIOP/20\right)\;+\;2.35\;\times \;\left(CCT/545\right)\\ 2.0,\;for\;Ln\left(SP-HC\right)=1.11\;+\;1.02\;\times \left(fIOP/20\right)\;+\;2.55\;\times \;\left(CCT/545\right)\\ 3.0,\;for\;Ln\left(SP-HC\right)=1.33\;+\;1.05\;\times \left(fIOP/20\right)\;+\;2.54\;\times \;\left(CCT/545\right) \end{array} \end{array}$$

where fIOP is in mmHg and CCT is in microns. For intermediate values of *Ln*(*SP* − *HC*), interpolation between the values of fSSI could be performed. With this equation form, the RMS error was 8.83%.

### Validation of fSSI Against Clinical Data

**Figure 5:**
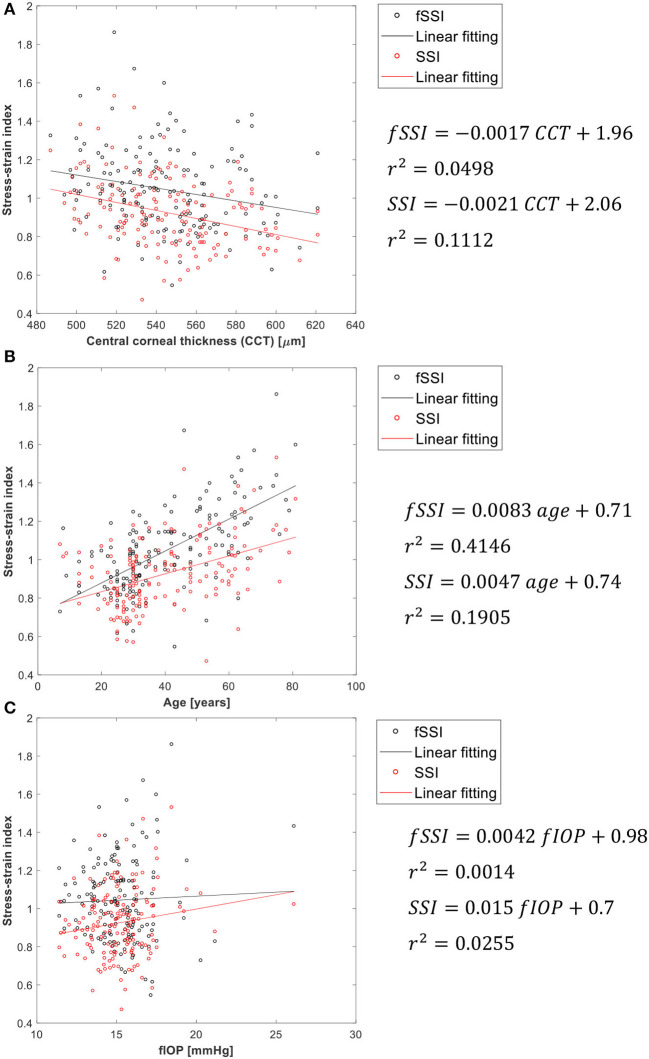
Association of the corneal material parameter fSSI with **(A)** CCT, **(B)** age, and **(C)** fIOP.

As additional validation of fSSI, its correlation with CCT, age and fIOP is assessed. Weak correlation with CCT and fIOP would be a sign of success along with positive correlation with age [where earlier evidence pointed at tissue stiffening with aging (Elsheikh et al., 2010a,b)]. The results shown in Figure 5 present better performance than SSI in maintaining weak correlation with CCT and IOP. Meanwhile, the correlation of fSSI with age was stronger than for SSI (*r*^2^ = 0.415 vs. 0.191). Moreover, as a validation against clinical corneal deformation profiles, six cases are presented in Figure S5 in terms of the spatial corneal deformations and temporal apical deformation.

## Discussion

The main objective of this study is to investigate the influence of considering the fluid-structure interaction between the air puff and the cornea on the reliability of methods to estimate the biomechanically-corrected IOP and the corneal material behavior. IOP is interlinked with material stiffness in a complex loop as the measurement of IOP in tonometry is commonly influenced by corneal stiffness, while the tangent modulus (a measure of stiffness) is known to increase with the level of IOP (Kirstein et al., 2011; Eliasy et al., 2019). The challenge to provide estimations of IOP and corneal stiffness that are independent of each other was dealt with in the present study using numerical modeling and employing the results to build algorithms to estimate fIOP and corneal material index fSSI. These algorithms included a number of Corvis deformation parameters, namely the first applanation pressure (AP1) and the highest concavity radius (R_HC_) in the fIOP equation, and the stiffness parameter (SP-HC) in the fSSI equation.

This challenge was addressed in earlier studies in the development of bIOP and SSI (Eliasy et al., 2018, 2019; Chen et al., 2019), and this study aimed to use more representative numerical modeling that considered the fluid-structure interaction between the air puff and the cornea. With this new model, which employed the arbitrary Lagrangian-Eulerian deforming mesh, small changes could be observed in the temporal and spatial pressure distribution profiles on the cornea, and these changes were dependent on the eye's geometric features and material stiffness. The FSI effect was more evident when the cornea's deformation was high as in cases small CCT or low IOP.

Consideration of these pressure distribution profiles in the development of an algorithm to estimate fIOP resulted in better performance compared to the bIOP in reducing the association of IOP measurements with both age and R, but maintained similar low correlation with CCT.

Consideration of the pressure distribution profiles in developing the fSSI algorithm resulted in similar improvements compared with the SSI with slightly weaker dependence on CCT and fIOP while maintaining similar correlation with age.

The development of these algorithms could benefit clinical practice in providing biomechanically-corrected IOP measurements to improve glaucoma diagnosis and management. They can also help in keratoconus detection via increasing the effectiveness of existing biomechanical indices such as the Tomography and Biomechanical Index (TBI) (Ambrósio et al., 2017) and the Corvis Biomechanical Index (CBI) (Vinciguerra et al., 2016b), especially that the FSI effect is more evident in soft corneas such as those with keratoconus (Andreassen et al., 1980; Ye et al., 2015; Vinciguerra et al., 2016b).

There were some limitations in the current study, which are important to note. The eye model employed in the study did not include soft tissue filling the orbital space and surrounding the eye which gives the eye freedom to move backward. Moreover, clinically, the air puff shooting direction can be sometimes at an angle from the eye axis and a modification for the mesh was done to apply the air puff at an angle, but the problem is that it's not known how the air puff will hit the cornea in order to make a global correction which fits with all patients. Finally, the current study concentrated on developing the numerical model and the algorithms for healthy eyes and the next step is to extend the study for keratoconic eyes before and after crosslinking.

In conclusion, we developed novel algorithms for IOP and corneal material estimation *in-vivo* for healthy corneas by considering the fluid-structure interaction between the air-puff of the Corvis ST tonometer and the eye globe. The algorithms demonstrated slightly better performance than bIOP and SSI, contributing further to the reliability of these algorithms and assisting their application in clinical practice.

## Data Availability Statement

All datasets generated for this study are included in the article/Supplementary Material.

## Ethics Statement

The Institutional review board of the University of Liverpool ruled that approval was not obligatory for this record review study. However, the ethical standards set out in the 1964 Declaration of Helsinki and their revision in 2013 were observed and all patients provided informed written consent before using their de-identified data in research.

## Author Contributions

AEls: conceptualization and resources. OM, K-JC, and JW: data curation. OM: formal analysis, software, and writing—original draft. OM and AEli: project administration. VT and AEls: supervision. OM, BL, JW, AA, VT, and AEls: validation. AEli, K-JC, and VT: visualization. OM, AEli, BL, AA, VT, and AEls: writing—review and editing. All authors reviewed the paper and gave final approval.

## Conflict of Interest

AEls is a consultant for OCULUS Optikgeräte GmbH. The remaining authors declare that the research was conducted in the absence of any commercial or financial relationships that could be construed as a potential conflict of interest.
