# Supplementary material for: Fluid-Structure Interaction Based Algorithms for IOP and Corneal Material Behavior
Source: Front Bioeng Biotechnol. 2020 Aug 28;8:970. doi: 10.3389/fbioe.2020.00970 (PMC7483485; doi:10.3389/fbioe.2020.00970)
Supplement: Supplementary file 2 [file Table_2.DOCX]

**Supplementary material**

**Mesh sensitivity analysis for the FSI model**

Figure S1: Mesh dependence study on both domains of the FSI model, showing the apex deformation from the eye model and pressure on apex from the CFD model and the model running time.


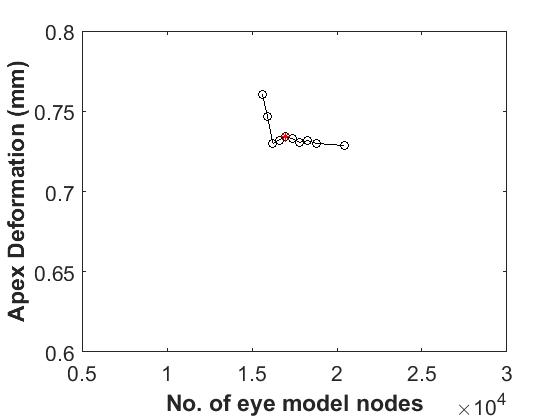

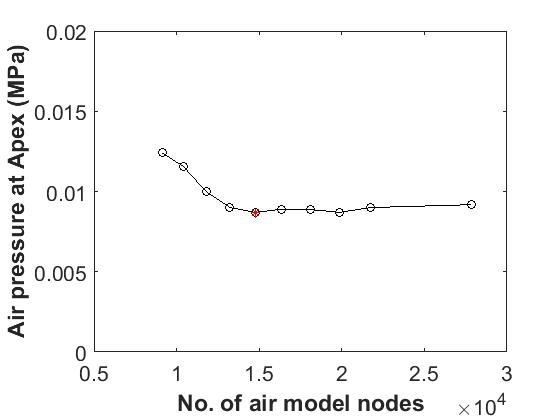

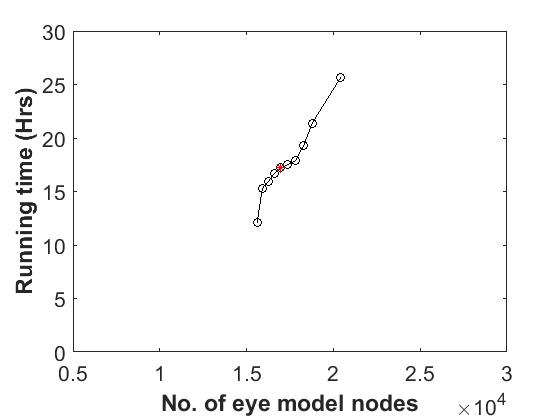


**Fluid-structure interaction (FSI) model of the air-puff test of the Corvis ST on full eye globe**


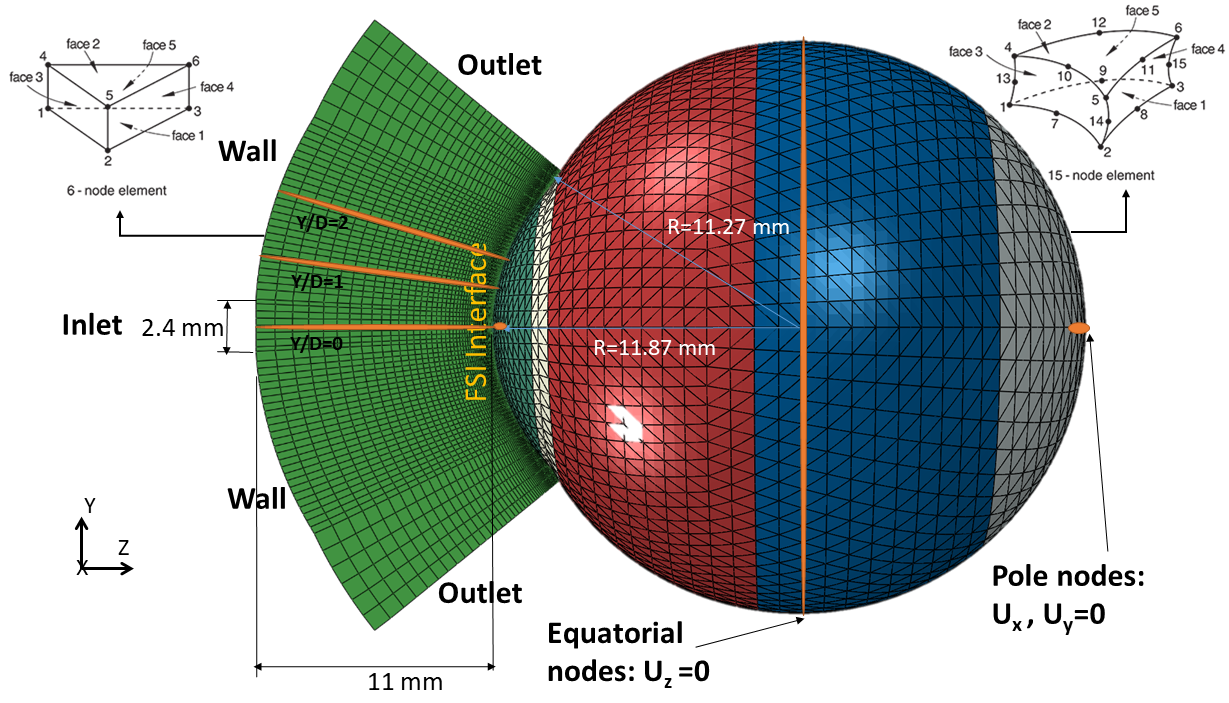


Figure S2: Geometry definition of the air puff and eye domains showing key dimensions, element types and boundary conditions. Ux , Uy , Uz are the deformations in the three dimensions, Maklad et al. [17]..


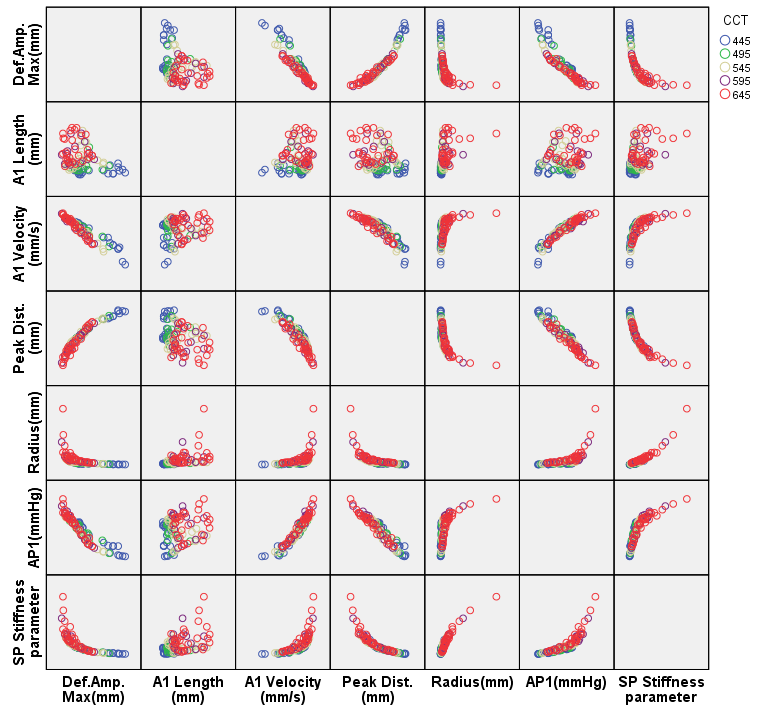
**Influence matrices of changing model input parameters on corneal response parameters**

(a)

(b)


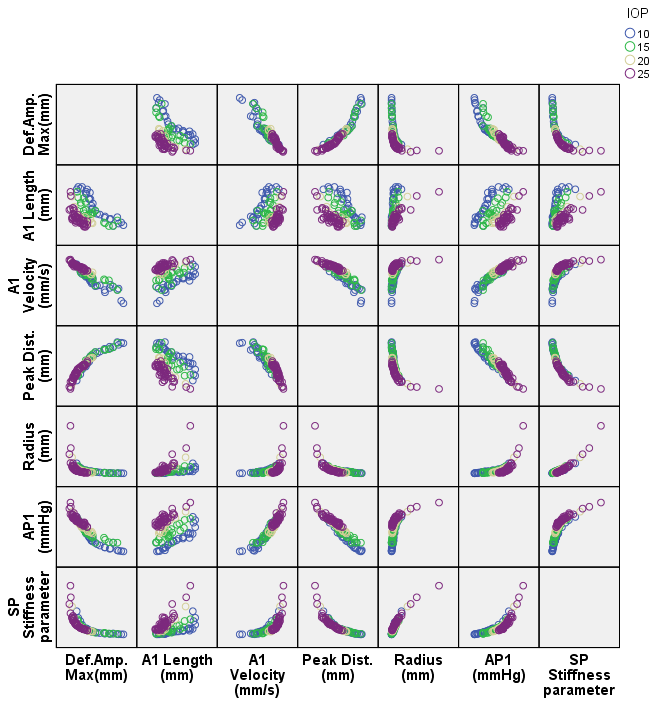

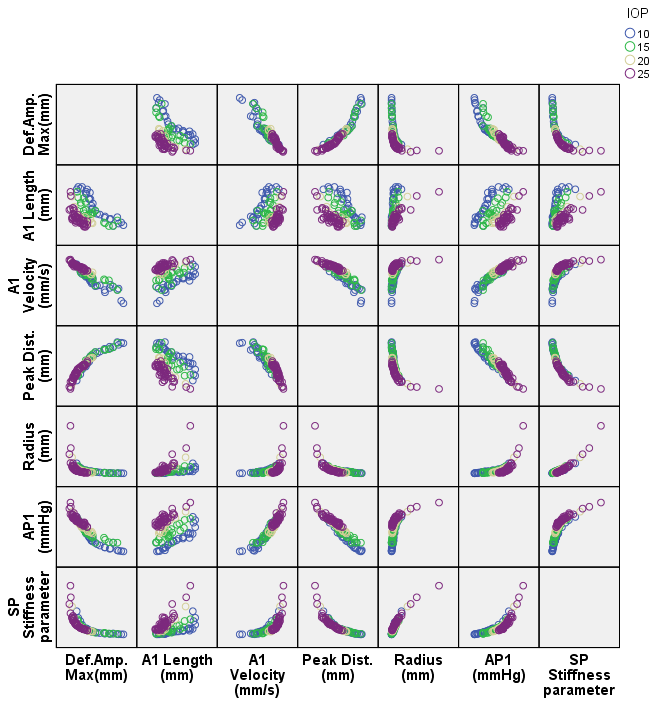


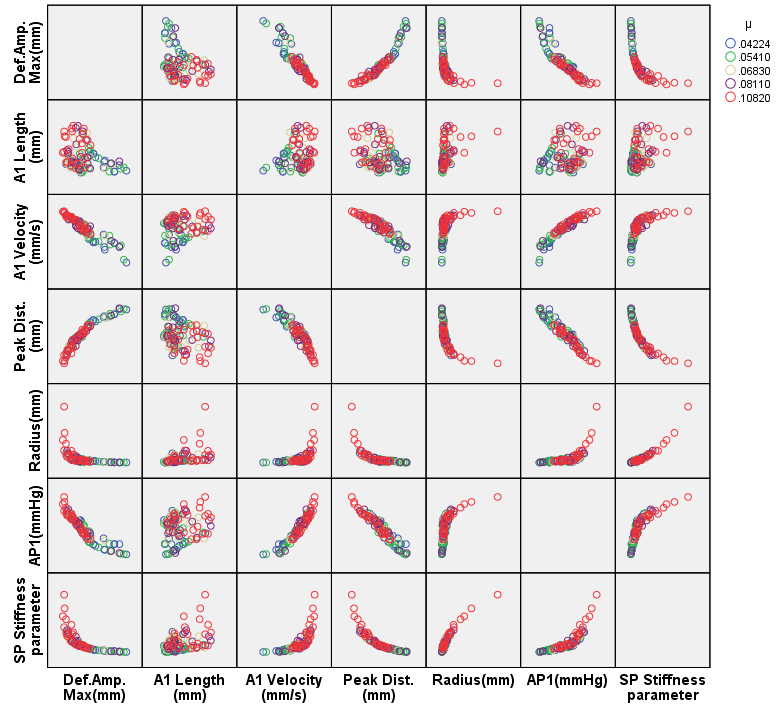

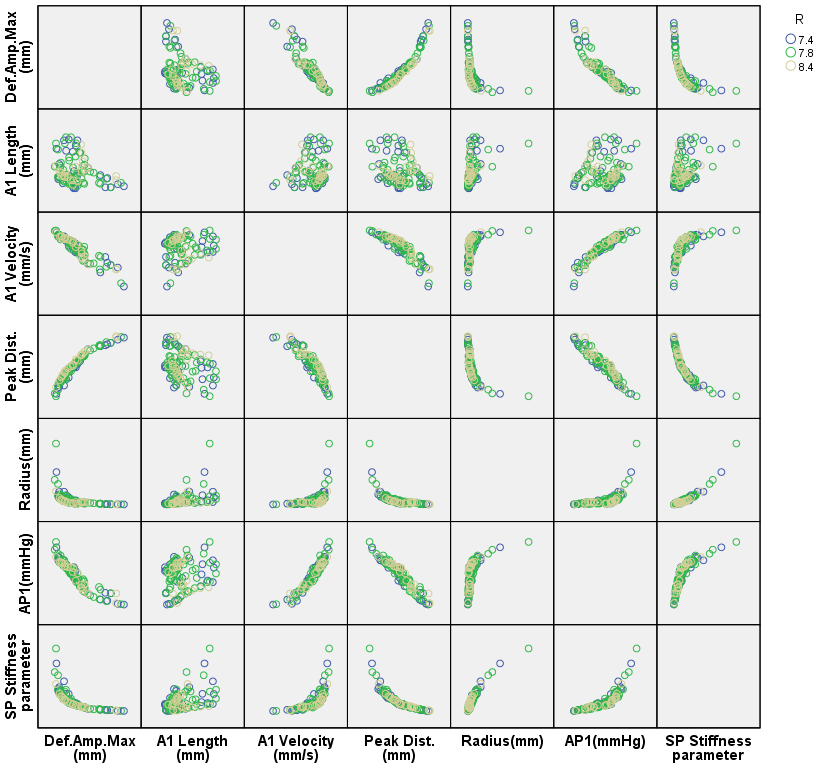


(d)

(c)

Figure S3: Influence matrices of changing (a) intraocular pressure (IOP), (b) central corneal thickness (CCT), (c) corneal material stiffness coefficient, and (d) central corneal curvature (R) on corneal response parameters.

Table S1: Correlation and relationship significance analysis between input and output parameters of the parametric study

| Variable | | A1 Time(ms) | A1 Length(mm) | A1 Velocity(mm/s) | HC Time(ms) | Peak Dist.(mm) | A1 Deformation Amp.(mm) | HC Deformation Amp.(mm) | AP1 (mmHg) | SP-HC Stiffness parameter |
| --- | --- | --- | --- | --- | --- | --- | --- | --- | --- | --- |
| IOP [mmHg] | Pearson Correlation (r) | .725^**^ | -.455^**^ | -.731^**^ | -.255^**^ | -.616^**^ | -.403^**^ | -.635^**^ | .736^**^ | .442^**^ |
|  | Sig. (2-tailed) | 0.000 | 0.000 | 0.000 | 0.007 | 0.000 | 0.000 | 0.000 | 0.000 | 0.000 |
| CCT [µm] | Pearson Correlation (r) | .382^**^ | .637^**^ | -.206^*^ | -0.122 | -.500^**^ | .673^**^ | -.493^**^ | .385^**^ | .468^**^ |
|  | Sig. (2-tailed) | 0.000 | 0.000 | 0.031 | 0.204 | 0.000 | 0.000 | 0.000 | 0.000 | 0.000 |
| μ | Pearson Correlation (r) | .338^**^ | .471^**^ | -.367^**^ | -.280^**^ | -.407^**^ | .432^**^ | -.377^**^ | .355^**^ | .434^**^ |
|  | Sig. (2-tailed) | 0.000 | 0.000 | 0.000 | 0.003 | 0.000 | 0.000 | 0.000 | 0.000 | 0.000 |
| R [mm] | Pearson Correlation (r) | -0.007 | -0.056 | -0.067 | 0.032 | 0.088 | -.253^**^ | -0.052 | 0.007 | -0.088 |
|  | Sig. (2-tailed) | 0.946 | 0.564 | 0.486 | 0.741 | 0.362 | 0.008 | 0.592 | 0.945 | 0.362 |

Note: IOP is intraocular pressure; CCT is central corneal thickness; μ is corneal material stiffness coefficient; R is corneal curvature radius; A1 is the first applanation; HC is the highest concavity; AP1 is the first applanation pressure; SP-HC is the stiffness parameter at highest concavity. **: Correlation is significant at the 0.01 level. *: Correlation is significant at the 0.05 level.

Figure S4: Analysis of air velocity, pressure coefficient and corneal deformation magnitude at the cornea. Plot (a) shows distribution of pressure coefficient reaching one at the centre representing the stagnation point and drops to zero at distance ~= 2 mm which indicates how the dynamic pressure converted into static pressure loading on the cornea. Plot(b) is more interesting, it shows distribution of the normalized axial velocity (V3/V3max) along the cornea showing zero at the centre, but negative values up to the 2 mm ring of the cornea, explaining the reflection of air away from corneal surface due to the impact, while Plot (c) is showing the deformation magnitude along the cornea with maximum value at the centre.

(a)

(b)

(c)

Temporal apical deformation

Spatial corneal deformations

Case 1

Case 2

Case 3

Case 4

Figure S5: Spatial corneal deformation and temporal apical deformation comparison, for the same six clinical cases in Figure 3 of the paper, after applying fIOP and fSSI estimation algorithms.

Temporal apical deformation

Spatial corneal deformations

Case 5

Case 6
