# Supplementary figures and images for: Fluid-Structure Interaction Based Algorithms for IOP and Corneal Material Behavior
Source: Front Bioeng Biotechnol. 2020 Aug 28;8:970. doi: 10.3389/fbioe.2020.00970 (PMC7483485; doi:10.3389/fbioe.2020.00970)

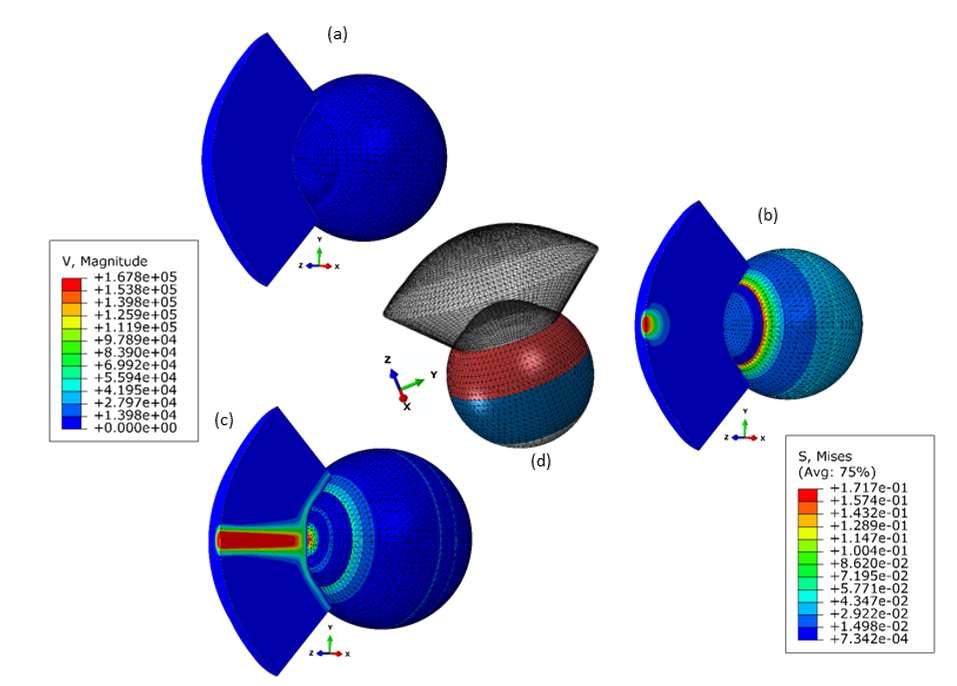

Supplement: Supplementary file 6 [file Data_Sheet_2.ZIP › Figures/F1.png]

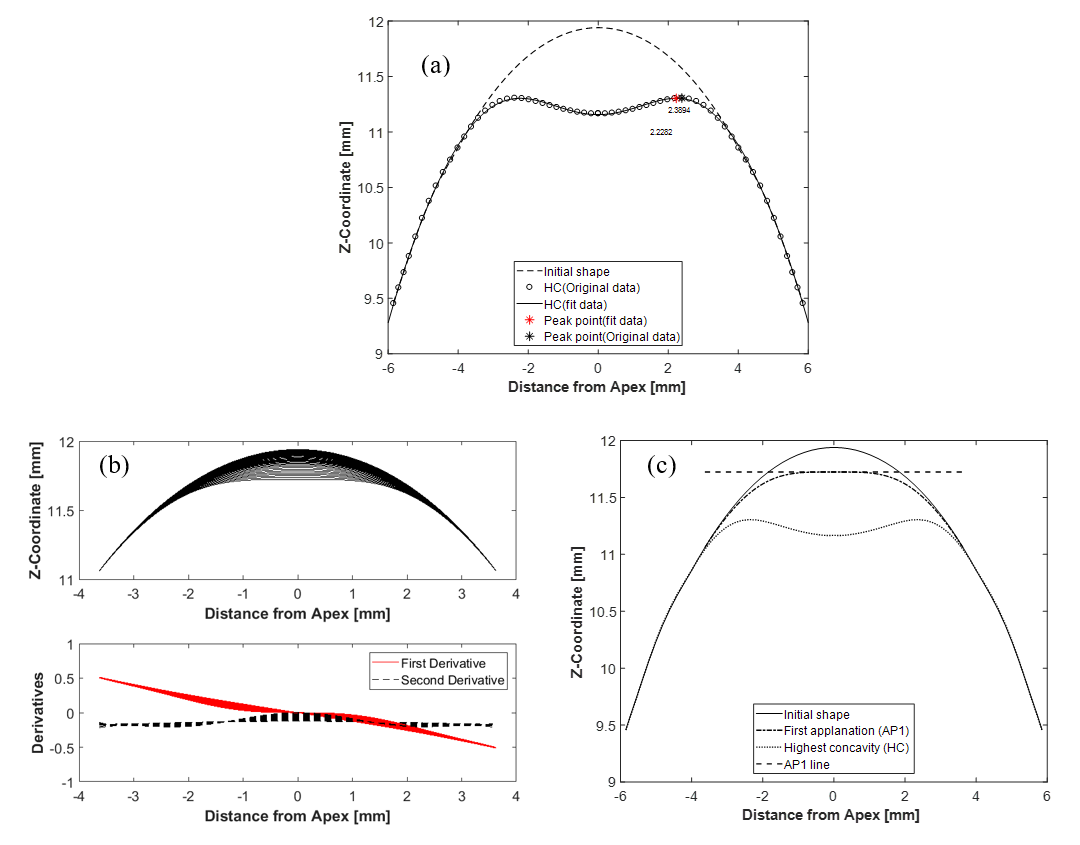

Supplement: Supplementary file 6 [file Data_Sheet_2.ZIP › Figures/F2.png]

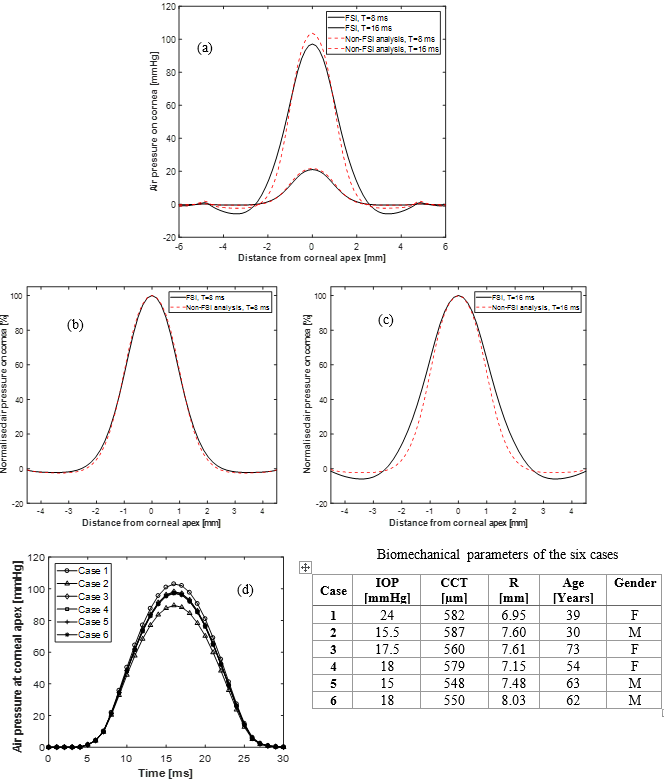

Supplement: Supplementary file 6 [file Data_Sheet_2.ZIP › Figures/F3.png]

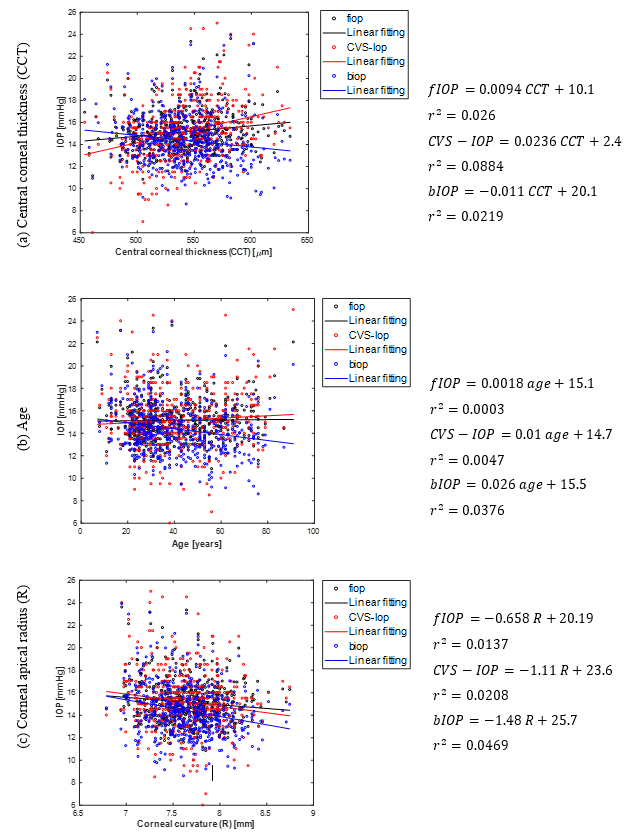

Supplement: Supplementary file 6 [file Data_Sheet_2.ZIP › Figures/F4.png]

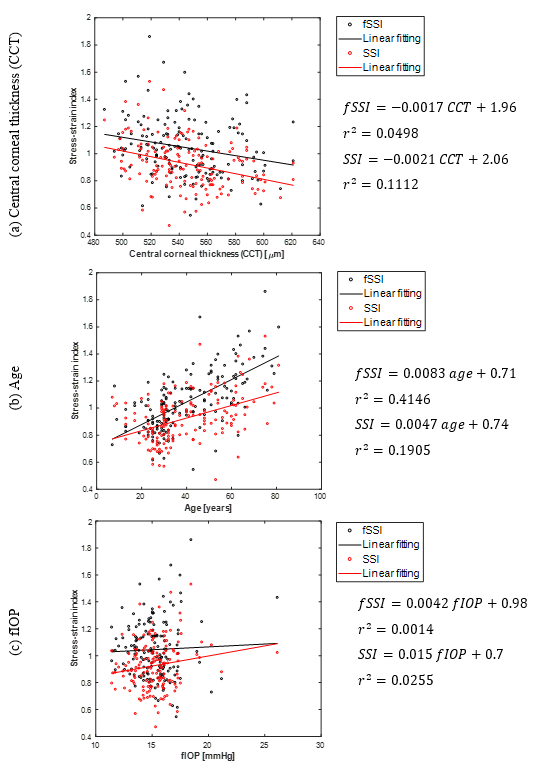

Supplement: Supplementary file 6 [file Data_Sheet_2.ZIP › Figures/F5.png]
